# Population Genomics of *Legionella longbeachae* and Hidden Complexities of Infection Source Attribution

**DOI:** 10.3201/eid2305.161165

**Published:** 2017-05

**Authors:** Rodrigo Bacigalupe, Diane Lindsay, Giles Edwards, J. Ross Fitzgerald

**Affiliations:** The Roslin Institute, University of Edinburgh, Midlothian, Scotland, UK (R. Bacigalupe, J.R. Fitzgerald);; Glasgow Royal Infirmary, Glasgow, Scotland, UK (D. Lindsay, G. Edwards)

**Keywords:** *Legionella longbeachae*, legionellosis, legionella, outbreaks, epidemiology, phylogeny, recombination, plasmids, bacteria, genomic diversity, environmental genotypes, pathogenic genotypes, source attribution

## Abstract

*Legionella longbeachae* is the primary cause of legionellosis in Australasia and Southeast Asia and an emerging pathogen in Europe and the United States; however, our understanding of the population diversity of *L. longbeachae* from patient and environmental sources is limited. We analyzed the genomes of 64 *L. longbeachae* isolates, of which 29 were from a cluster of legionellosis cases linked to commercial growing media in Scotland in 2013 and 35 were non–outbreak-associated isolates from Scotland and other countries. We identified extensive genetic diversity across the *L. longbeachae* species, associated with intraspecies and interspecies gene flow, and a wide geographic distribution of closely related genotypes. Of note, we observed a highly diverse pool of *L. longbeachae* genotypes within compost samples that precluded the genetic establishment of an infection source. These data represent a view of the genomic diversity of *L. longbeachae* that will inform strategies for investigating future outbreaks.

Legionellosis presents as 2 clinically distinct forms: an influenza-like illness called Pontiac fever and a severe pneumonia known as Legionnaires’ disease (*1*). In Europe and the United States, most legionellosis cases are caused by *Legionella pneumophila* serogroup 1 (*1*,*2*); <5% of cases are caused by nonpneumophila *Legionella* spp. (*3*,*4*). In Australasia, New Zealand, and some countries in Asia, infections caused by *L. longbeachae* occur at comparable levels to infections caused by *L. pneumophila* (*5*–*7*). Unlike *L. pneumophila* infections, which are typically linked to artificial water systems, *L. longbeachae* infections are associated with exposure to soil, compost, and potting mixes (*8*).

The number of legionellosis cases caused by *L. longbeachae* is increasing worldwide (*7*), with a notable rise reported across Europe (*9*–*11*). Within the United Kingdom, most *L. longbeachae* infections have been identified in Scotland, where 6 cases were diagnosed during 2008–2012 (*12*) and another 6 were diagnosed in the summer of 2013 and represented a singular increased incidence or cluster with all patients requiring intensive care hospitalization (*11*). Epidemiologic investigation revealed that most patients from the 2013 cluster were avid gardeners, and *L. longbeachae* was isolated from respiratory secretions and from samples of the growing media they had used for gardening before becoming ill (*11*,*12*). However, an investigation into the provenance of the growing media did not reveal a single commercial or manufacturing source that would suggest a common origin for the *L. longbeachae* associated with the outbreak (*11*).

Molecular typing methods used to discriminate between *L. longbeachae* and other *Legionella* spp. and between the 2 *L. longbeachae* serogroups have limited efficacy, and although considerable evidence supports growing media as a source for *L. longbeachae* infections (*13*,*14*), there is still a lack of genetic evidence for an epidemiologic link. Furthermore, a population genomic study involving large numbers of *L. pneumophila* isolates has been conducted (*15*,*16*), but the same has not been done for *L. longbeachae*, so the diversity of environmental and pathogenic genotypes and the relationship between them remains unknown for *L. longbeachae*. To examine the etiology of the 2013 cluster of legionellosis cases in Scotland in the context of *L. longbeachae* species diversity, we analyzed the genomes of 70 *Legionella* spp. isolates from 4 countries over 18 years.

## Materials and Methods

### Bacterial Isolates

We sequenced 65 isolates that had previously been identified as *L. longbeachae*. These isolates were obtained during 1996–2014 from several patients, growing media samples (including compost and soil), and a hot water supply. Of these isolates, 55 were from Scotland (29 from the 2013 cluster of infections and 26 from other clinical and environmental samples) and 10 were from patients and environmental compost samples in New Zealand (Technical Appendix Table). 

In our analysis, we also included all publicly available genome sequences for *L. longbeachae*: *L. longbeachae* NSW150 (serogroup 1) and *L. longbeachae* C-4E7 (serogroup 2) isolated from patients in Australia; and *L. longbeachae* D-4968 (serogroup 1), *L. longbeachae* ATCC39642 (serogroup 1), and *L. longbeachae* 98072 (serogroup 2) isolated from patients in the United States (*17**–**19*). We sequenced multiple isolates (n = 2 to 5) for each of 3 patients and their linked growing media samples from the 2013 outbreak in Scotland and for 2 additional compost samples. The species of all isolates had been determined by serotyping or macrophage infectivity potentiator (mip) gene sequencing (*20**,**21*).

### Bacterial Culture, Genomic DNA Isolation, and Whole-Genome Sequencing

We cultured *Legionella* spp. isolates in a microaerophilic and humid environment at 37°C on BCYE (buffered charcoal yeast extract) agar plates for 48 h. We then picked individual colonies from the plates and grew them in ACES-buffered yeast extract broth containing *Legionella* BCYE Growth Supplement (Oxoid Ltd., Basingstoke, UK) with shaking at 37°C for 24–48 h. We extracted genomic DNA from fresh cultures by using the QIAGEN DNeasy Blood and Tissue Kit (QIAGEN Benelux B.V., Venlo, the Netherlands).

We prepared sequencing libraries by using the Nextera XT kit for MiSeq or HiSeq (all from Illumina, San Diego, CA, USA) sequencing at Edinburgh Genomics, University of Edinburgh (Edinburgh, Scotland, UK). For each isolate, one 2 × 250–bp or two 2 × 200–bp paired-end sequencing runs were carried out using the MiSeq and HiSeq technologies, respectively. Raw reads were quality checked using FastQC v0.10.1 (*22*), and primers were trimmed by using Cutadapt (*23*). We used wgsim software (*24*) to simulate sequence reads for publicly available, complete whole-genome sequences.

### Bioinformatic Analysis and Data Deposition

A detailed description of the bioinformatic analyses is available in the online Technical Appendix. The sequence data for the 65 genomes of *Legionella* spp. sequenced in this study were deposited in the SRA database (accession no. PRJEB14754).

## Results

### Limitations of Current Typing Approaches for *Legionella* spp. Identification

**Figure 1:**
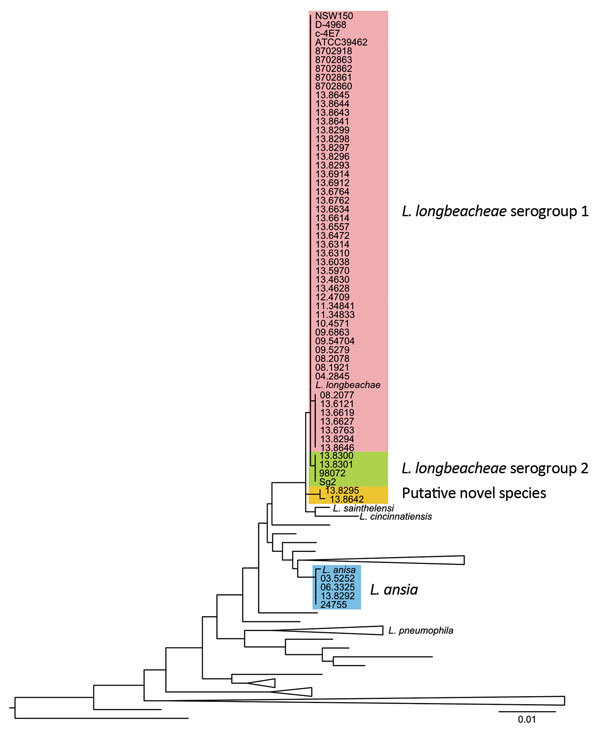
16S rRNA gene–based phylogenetic tree of the sequenced genomes and all the cultured and type *Legionella* spp. strains available in the ribosomal database project (http://rdp.cme.msu.edu/), as accessed in May 2015. Scale bar indicates the mean number of nucleotide substitutions per site.

We sequenced 65 isolates obtained from several patients and environmental samples over 18 years in different countries and previously identified as *L. longbeachae*. To confirm the species identity of the *Legionella* isolates, we constructed a phylogenetic tree that included all *Legionella* type strains for which cultures are available, based on the 16S rRNA gene sequence (*25*). We also built phylogenetic trees based on the whole-genome content and core-genome diversity. For each approach, 64 of the 70 isolates examined co-segregated within the *L. longbeachae*–specific clade, 4 isolates clustered with *Legionella anisa*, and 2 belonged to a separate clade that was distinct from all known *Legionella* spp. (Figure 1; Technical Appendix Figures 1, 2). The species identities were further supported by determination of the average nucleotide identity values (Technical Appendix Figure 3), a widely used method for bacterial species delineation based on genomic relatedness (*26*). Of note, *L. anisa* is the most common nonpneumophila *Legionella* spp. in Europe (*27*–*29*). In addition, *L. longbeachae* isolates 13.8642 (from a compost sample from Scotland) and 13.8295 (from a patient in New Zealand) belong to a putative novel *Legionella* spp. Overall, the data indicate that current serotyping methods and *mip* gene sequencing are limited in their capacity to identify *L. longbeachae* to the species level.

To investigate the genetic relatedness of *L. longbeachae* strains associated with the 2013 outbreak to temporally and geographically distinct isolates, we constructed a core genome–based neighbor-joining tree of the 64 confirmed *L. longbeachae* isolates obtained from 4 countries over 18 years (Technical Appendix Figure 4). This phylogenetic tree presents a comet-like pattern, with 2 distinct clades separated by 9,911 single-nucleotide polymorphisms, representing the major serogroups (serogroups 1 and 2) previously identified for *L. longbeachae* (*20*), each containing isolates from patient and environmental samples from different years. In contrast with findings from a previous analysis of 2 isolates of *L. longbeachae* serogroup 1 (*20*), we observed a higher diversity among the 56 isolates within serogroup 1 (Technical Appendix Figures 1, 4); this finding is not unexpected, given the difference in the number of genomes examined. Nevertheless, compared with isolates from the same serogroup in other *Legionella* spp., such as *L. pneumophila* serogroup 1 (2% polymorphism) (*20*), *L. longbeachae* serogroup 1 exhibits a lower diversity (<0.1% polymorphism). Although serogroup 1 and 2 clades contained isolates from Scotland, Australasia, and the United States, 96% of the isolates from Scotland (including all of the 2013 outbreak isolates) belonged to serogroup 1, suggesting that serogroup 1 may be more clinically relevant in Scotland than in some other countries where *L. longbeachae* is a more established cause of legionellosis. However, analysis of more isolates from different countries would be required to investigate this observation further.

### Effect of Recombination on *L. longbeachae* Serogroup 1 Population Structure

**Figure 2:**
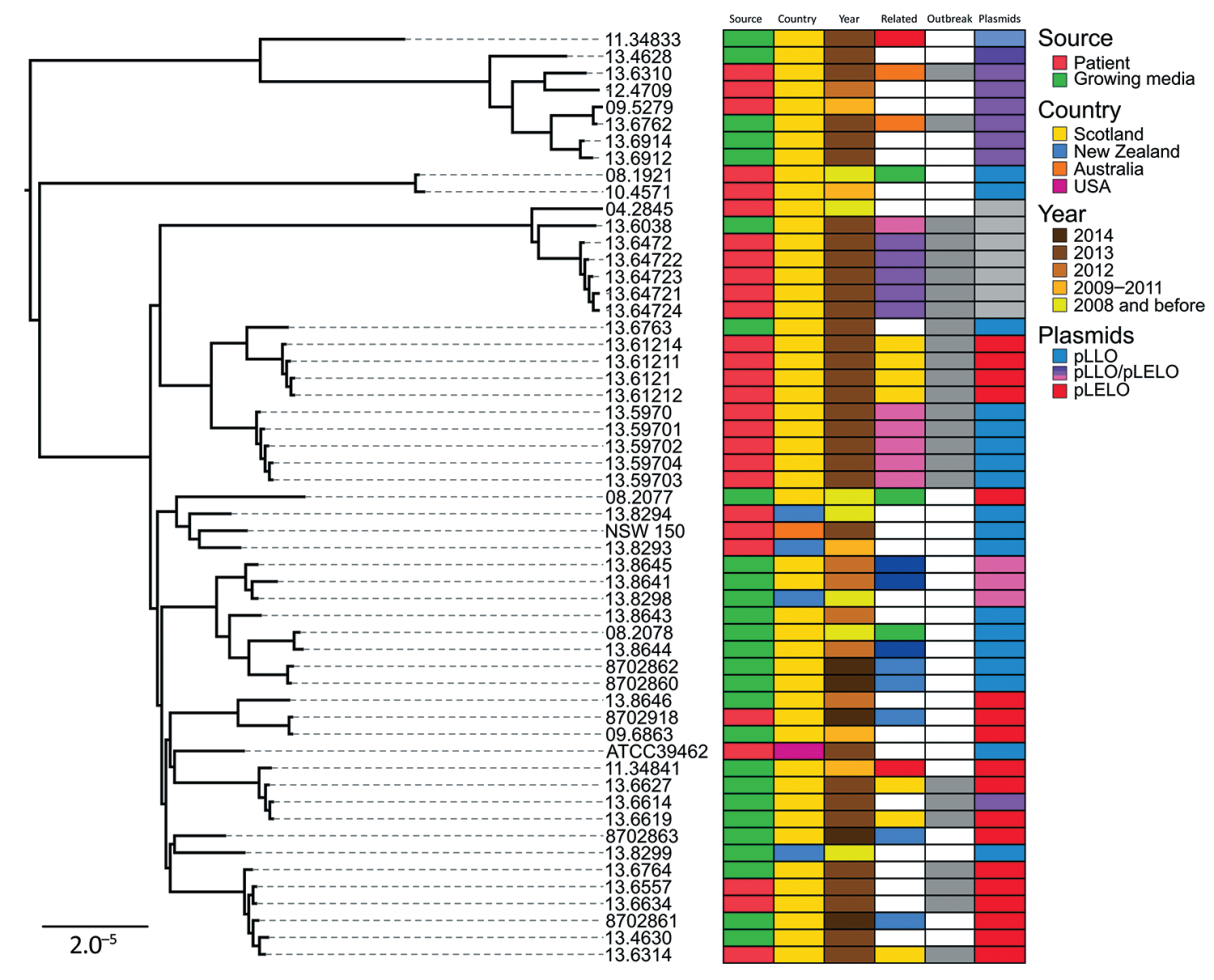
Core genome–based maximum-likelihood phylogeny of *Legionella longbeachae* serogroup 1 isolates corrected for recombination; source, country, year of isolation, relatedness and plasmid carriage are indicated. Related isolates are shown in the same color; those from the 2013 outbreak are indicated by gray. Isolates from the same patient are clustered together but do not co-segregate with cognate compost samples. Scale bar indicates the mean number of nucleotide substitutions per site.

It is established that recombination has played a key role in shaping the evolutionary history of *L. pneumophila,* but its effect on *L. longbeachae* population structure is unknown (*22*,*30*). This knowledge is critical because for highly recombinant bacteria, recombination networks may represent evolutionary relationships more explicitly than traditional phylogenetic trees. Therefore, we constructed a recombination network of all serogroup 1 isolates by using the neighbor-net algorithm of SplitsTree4 (*31*). The resultant network displayed a reticulate topology with an extensive reticulated background from which clusters of isolates emerge, supporting an evolutionary history involving recombination (p< 0.01 by ϕ test) (*32*), followed by clonal expansion and subsequent additional recombination events among some lineages (Technical Appendix Figure 5). Using BratNextGen (*33*), we identified a total of 94 predicted recombination events affecting more than half of the core genome (1.74 Mb of 3.36 Mb) and representing recent and ancient recombination events of different sizes (range 1,350 bp–350 Kbp) distributed across the phylogeny (Technical Appendix Figure 6). Given the reported limitation in sensitivity of BratNextGen for the identification of all recombination events (*34*), we also used ClonalFrameML (*35*), an algorithm that uses maximum likelihood inference to simultaneously detect recombination in bacterial genomes and account for it in phylogenetic reconstruction. The estimated average length of the recombined fragments was 8,047 bp, and the ratio of recombination to mutation was 1.42, indicating a greater role for recombination over mutation in the diversification of *L. longbeachae*. This estimate is in accordance with early estimates for *L. pneumophila* based on multiple gene sequence data (*36*), but it is low compared with recent estimates based on whole-genome sequence data [recombination to mutation ratios of 16.8 (*30*) or 47.93 (*37*)]. Differences in the clonal diversity of *Legionella* spp. sequence datasets used to determine recombination rates could affect the estimates. Reconstruction of the phylogeny after removal of all predicted recombinant sequences resulted in a tree with largely similar clusters of isolates but with reduced branch lengths and variation in the position of nodes deep in the phylogeny (Figure 2).

### Accessory Genome Analysis Indicates Extensive Interspecies and Intraspecies Gene Flow

**Figure 3:**
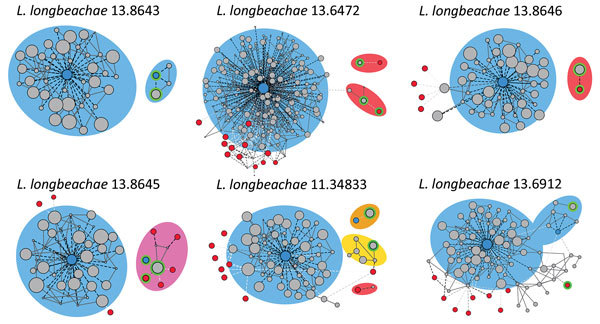
*Legionella longbeachae* plasmid analysis: contigs networks reconstructions for 6 representative *L. longbeachae* types of plasmid content. The networks of the contigs representing the main chromosome and plasmids comprising the genome obtained by using PLACNET (*38*), a program enabling reconstruction of plasmids from whole-genome sequence datasets. The sizes of the contig nodes (in gray) are proportional to their lengths; continuous lines correspond to scaffold links. Dashed lines represent BLAST (https://blast.ncbi.nlm.nih.gov/Blast.cgi) hits to the *L. longbeachae* (blue) or *L. pneumophila* (red) strains; intensity of the line is proportional to the hit (white indicates low, black indicates high). Green lines correspond to plasmid contigs. Background colors indicate species relatedness for the main chromosome and plasmids (blue for *L. longbeachae*, red for *L. pneumophila*, pink for a combination of both, and yellow for previously unidentified genomic content).

The extent to which horizontal gene transfer occurs among *L. longbeachae* isolates and between *L. longbeachae* and other *Legionella* spp. is unknown. In our study, the pangenome of *L. longbeachae* represented by the 56 serogroup 1 isolates was 6,890 genes, including a core genome of 2,574 genes; the average gene content was 3,558 genes per strain. The accessory genome, which included only strain-dependent genes varied from 809 to 1,155 genes, depending on the strain. A parsimony clustering analysis based on the presence or absence of all genes classified the isolates in a manner distinct from that in a core genome–based maximum-likelihood tree, suggesting extensive horizontal gene transfer among *L. longbeachae* isolates (Technical Appendix Figures 1, 2). BLAST (https://blast.ncbi.nlm.nih.gov/Blast.cgi) analysis of all assembled contigs was used to filter for plasmid-related homologous sequences, revealed 2 major plasmids: pLLO, described previously in *L. longbeachae* NSW150 (*20*), and pLELO, originally identified in *L. pneumophila subsp. pneumophila* (*22*). Of the 55 serogroup 1 isolates, 36 contained sequences for the pLLO and pLELO plasmids. Of note, the distribution of these plasmids among the *L. longbeachae* isolates correlated with the gene content–based clustering, whereas the distribution of plasmids in the core genome–based tree was independent of the phylogeny (Figure 2). In addition, 11 isolates appeared to contain plasmids with sequences homologous to those for pLLO and pLELO, which is indicative of recombinant forms of the plasmid. Further examination of plasmid diversity using a modified version of PLACNET (*38*), a program enabling reconstruction of plasmids from whole-genome sequence datasets, confirmed that some plasmids consisted of a mosaic of recombinant fragments homologous to pLELO, pLLO, or other unknown plasmids (Figure 3). Overall, these data indicate the high prevalence of specific plasmids among *L. longbeachae* isolates and reveal extensive recombination and horizontal gene transfer among different *Legionella* spp (*39*). The high prevalence of plasmids in *L. longbeachae* is notable, considering these elements may be less common in *L. pneumophila* (*30*).

**Figure 4:**
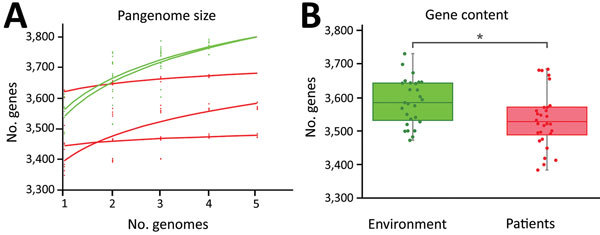
Variation in gene content between environmental and patient *Legionella longbeachae* samples. A) Increase in pangenome size with every addition of a *L. longbeachae* genome. Environmental isolates pangenomes (green) are larger and continue increasing after the addition of 5 genomes, consistent with an open pangenome, but the within-patient pangenome plateaus quickly, consistent with a more closed pangenome. B) Average gene content of environmental isolates is significantly higher than that of clinical isolates (p = 0.01474).

To examine the possibility that clinical and environmental isolates of *L. longbeachae* contained genomic differences reflecting their distinct origins, we compared their accessory genome content. For isolates obtained from a single patient sample, the accessory genome was highly conserved compared with those for environmental isolates from a single compost sample or closely related environmental isolates from distinct compost samples (Figure 4, panel A). In addition, considering the average gene content of all sequenced isolates (28 clinical and 27 environmental), the gene content for *L. longbeachae* from growing media samples (3,586 genes) was significantly higher than that for isolates from patients (3,533 genes; 2-sample *t*-test, *t* = 2.5213; d.f. = 53; p = 0.01474) (Figure 4, panel B). The data imply that gene loss occurs during human infection or that *L. longbeachae* strains with reduced gene content have enhanced human infectivity. However, we did not identify a specific enriched gene or functional category in clinical or environmental samples (data not shown).

### Source Attribution Confounded by Complex Serogroup 1 Populations within Environmental Samples

Having accounted for the influence of recombination on the phylogeny of *L. longbeachae*, we investigated the diversity of isolates associated with 5 patients and their linked compost samples obtained during 2008–2014, including 3 patients from the 2013 outbreak in Scotland. Of note, isolates from the 2013 outbreak were distributed across several subclades of the tree, indicating that the infections were caused by different strains (Figure 2). However, all isolates from a single patient clustered together, consistent with a monoclonal etiology of each infection. Of note, for all 5 patients, clinical isolates were not closely allied to the environmental isolates obtained from linked compost samples, and therefore a genetic link between patient and compost samples could not be established. Most subclades included isolates of diverse geographic origin, consistent with a wide distribution for *L. longbeachae* strains; however, 3 *L. longbeachae* isolates originating from Australasia (strains 13.8294, 13.8293, and NSW150) belonged to their own region-specific cluster (Figure 2).

We hypothesized that the lack of genetic relatedness between *L. longbeachae* isolates from patients and linked compost samples could be explained by a highly diverse population of *L. longbeachae* in growing media samples compounded by a sampling strategy consisting of a single sequenced isolate. All 5 compost samples for which we had >1 isolate contained isolates distributed across multiple clades in the phylogenetic tree. In particular, 5 isolates from the same growing media sample linked to a patient infected in Edinburgh in 2014 were distributed across 4 distinct clades, demonstrating that within a single environmental sample, considerable species diversity may be represented (Figure 2). Taken together, these data suggest that for future outbreak investigations, extensive sampling of environmental samples may be required to identify genotypes responsible for episodes of legionellosis infection, if indeed they are present.

## Discussion

Our findings reveal the population genomic structure for *L. longbeachae*, an emerging pathogen in Europe and the United States, and includes a genome-scale investigation into an outbreak of *L. longbeachae* legionellosis. We provide evidence for extensive recombination and lateral gene transfer among *L. longbeachae*, including the presence of widely distributed mosaic plasmids that have likely recombined with plasmids from other *Legionella* spp., suggesting an ecologic overlap or shared habitat. Our analysis highlights the need to account for recombination events when determining the genetic relatedness of *L. longbeachae* isolates.

Our application of whole-genome sequencing for diagnostic purposes revealed the misidentification, using current serotyping methods, of several *L. anisa* isolates as *L. longbeachae* and led to the identification of a putative novel *Legionella* sp. linked to legionellosis. These findings highlight the limitations of current typing methods for differentiation of *Legionella* spp. and accurate identification of legionellosis etiology.

We used whole-genome sequencing to attempt to establish a genetic link between legionellosis infections and associated compost samples. Our inability to establish a link probably reflects the traditional strategy of single isolate sampling, which when applied to a highly diverse pool of *L. longbeachae* genotypes fails to detect the infecting genotype. We suggest that the approach to investigating the source of future legionellosis cases linked to growing media will require a radical revision of sampling protocols to maximize the chances of isolating the infecting strain, if present. Taken together, our findings provide a view of the population structure of *L. longbeachae* and highlight the complexities of tracing the origin of legionellosis associated with growing media. Overall, our findings demonstrate the resolution afforded by whole-genome sequencing for understanding the biology underpinning legionellosis and provide information that should be considered for future epidemiologic investigations.

Technical Appendix Description of the bioinformatic analysis in a study of the hidden complexities of source attribution for *Legionella longbeachae* infections revealed by population genomics.
