# Supplementary material for: Population Genomics of Legionella longbeachae and Hidden Complexities of Infection Source Attribution
Source: Emerg Infect Dis. 2017 May;23(5):750–7. doi: 10.3201/eid2305.161165 (PMC5403047; doi:10.3201/eid2305.161165)
Supplement: Technical Appendix — Description of the bioinformatic analysis in a study of the hidden complexities of source attribution for Legionella longbeachae infections revealed by population genomics. [file 16-1165-Techapp-s1.pdf]

Article DOI: http://dx.doi.org/10.3201/eid2305.161165

Hidden Complexities of Source Attribution for *Legionella longbeachae* Infections Revealed by Population Genomics

Technical Appendix

Materials and Methods

Genome Assemblies and Variant Calling

De novo assemblies of the *Legionella* isolates were produced using SPAdes 2.5.1. (*1*) (k values of 21, 33, 55, 77, 99 and 127), generating a median of 106 contigs per genome (range, 38–402 contigs), with an average of 4.16 Mb in length (3.98–4.52 Mb) and an average N50 of 130 Kb (29 Kb-291 Kb).

The error-corrected reads produced by SPAdes were mapped against the *Legionella longbeachae* reference genome of strain NSW 150 (GenBank accession number NC_013861) using BWA 0.5.9 (*2*) with default parameters. SNPs were called using Samtools 1.18 (*3*) and those absent in at least 30% of the reads, with quality below 30 and depth below 3 were filtered out. The output from this filtering was used to construct consensus genomes of all the isolates for further phylogenetic analyses.

Analysis of Genome Content

The contigs were annotated using Prokka v1.10 (*4*) and orthologous genes were clustered using the algorithm OrthoMCL (*5*) integrated in the software Get_homologs (*6*). We selected the options –f 50 (filters by 50% length difference within clusters) and –t 0 (for reporting all the clusters), resulting in 1801 core genome clusters. This program was also run using the Sg1 isolates only as input, specifying the options minimum percentage coverage (-C 80) and percentage identity (-S 85), which generated a core genome of 2574 gene clusters.

We also used JSpecies (*7*) to compute the average nucleotide identity values (BLAST; ANIb) between several pairs of isolates. These ANIb results were represented on a plot where isolates were clustered according to the 16S rRNA phylogenetic tree. In addition, a pangenomic

tree of the OMCL binary matrix from get_homologs.pl using all the *Legionella* isolates was constructed using the compare_clusters.pl script.

Evolutionary and Phylogenetic Analysis

To confirm the identity of the isolates, a Neighbor-Joining tree based on the 16S rRNA gene of the sequenced genomes and all the cultured type *Legionella* strains available in the Ribosomal Database Project (*8*) (as of 01/06/2015) was constructed. The RNAmmer 1.2 server (*9*) was used to identify the 16S rRNA genes in the de novo assemblies, which were then aligned using MUSCLE with default parameters (*10*). The Neighbor-Joining tree was estimated using the Hasegawa–Kishino–Yano model and 1000 bootstrap resampling replicates using the program Geneious 5.4.6 (*11*).

To construct a phylogeny based on the whole genome sequence data, the 1801 orthologous open reading frames identified using OrthoMCL were aligned using MUSCLE 3.8.31 (*10*). Individual protein alignments were translated back to DNA alignments using pal2nal v14 (*12*) and the resultant alignments were concatenated using catfasta2phyml.pl (*13*) into an 1110024 bp long super-alignment. A ML phylogenetic tree was estimated based on this alignment using RAxML. C-4E7 was excluded from the original clustering as the low quality of the assembly significantly reduced the size of the core genome.

The *L. longbeachae* phylogeny was reconstructed using a Neighbor-Joining approach in Splitstree4 (*14*). Phylogenies of *L. longbeachae* Sg1 isolates before and after removing recombination were reconstructed from the genome alignments using RAxML 7.2.6 (*15*).

Detection of Recombination

Recombination was examined using the SplitsTree4 program (version 4.13.1) (*14*). A phylogenetic network was computed on the *L. longbeachae* Sg1 multiple genome alignment using the Neighbor-Net method implemented in this software. The statistical significance of the tree was confirmed using a Phi test (*16*). Recombination was detected on the core genome alignment of the Sg1 isolates using BratNextGen (*17*). After drawing a PSA tree, we selected a cutoff of 0.042, which split the tree into 8 clusters. We used 20 iterations for the recombination learning algorithm and after performing 100 replicate runs in a single processor we selected a threshold of 5% for estimating the significance of recombination. Finally, the *L. longbeachae* Sg1 ML tree and the whole genome alignment were used as input for ClonalFrameML (*18*) to generate a phylogeny with branch lengths corrected for recombination. 100 pseudo-bootstrap

replicates were used to estimate the uncertainty in the EM model and the option -ignore_user_sites with the list of non-core coordinates was parsed.

Plasmid Analysis

We used PLACNET, a software that constructs a network of contigs interactions, for the identification and visualization of plasmids (*19*). Bowtie2 v2.0.6 (*20*) was first used to find all possible scaffold links of the contigs by mapping the reads to them. Length and insert sizes of the reads mapped were calculated using Picard-Tools v1.90 (*21*). These files and metrics were parsed as input for placnet.pl, which produced a plasmid network from which we extracted the scaffolds. We then performed a BLAST search of the contigs assembly files to a database containing all the bacteria and plasmids genomes available in the NCBI ftp site (*22*) (created in March 2015) and results were filtered as follows: contigs longer than 200 bp, with a bitscore below 1e-26 and that had at least a blast hit over 5% of the contig size. The results were further analyzed to classify the nodes into one of these categories: "hit completely to a single reference genome," "split nodes that hit to a single reference genome" and "nodes that hit several genomes." The hits and the scaffolds were combined into a network that was uploaded into Cytoscape (*23*). Recommendations given in the PLACNET manual were followed to visualize the chromosome and plasmids networks. BLAST was finally used to search for *Legionella* spp. plasmid related sequences in the contigs.

References

1. Bankevich A, Nurk S, Antipov D, Gurevich AA, Dvorkin M, Kulikov AS, et al. SPAdes: a new genome assembly algorithm and its applications to single-cell sequencing. J Comput Biol. 2012;19:455–77.

2. Li H, Durbin R. Fast and accurate long-read alignment with Burrows–Wheeler transform. Bioinformatics. 2010;26:589–95.

3. Li H, Handsaker B, Wysoker A, Fennell T, Ruan J, Homer N, et al. 1000 Genome Project Data Processing Subgroup. The Sequence Alignment/Map format and SAMtools. Bioinformatics. 2009;25:2078–9.

4. Seemann T. Prokka: rapid prokaryotic genome annotation. Bioinformatics. 2014;30:2068–9.

5. Li L, Stoeckert CJJ Jr, Roos DS. OrthoMCL: identification of ortholog groups for eukaryotic genomes. Genome Res. 2003;13:2178–89.

6. Contreras-Moreira B, Vinuesa P. GET_HOMOLOGUES, a versatile software package for scalable and robust microbial pangenome analysis. Appl Environ Microbiol. 2013;79:7696–701.

7. Richter M, Rosselló-Móra R. Shifting the genomic gold standard for the prokaryotic species definition. Proc Natl Acad Sci U S A. 2009;106:19126–31.

8. Cole JR, Wang Q, Fish JA, Chai B, McGarrell DM, Sun Y, et al. Ribosomal Database Project: data and tools for high throughput rRNA analysis. Nucleic Acids Res. 2014;42(D1):D633–42.

9. Lagesen K, Hallin P, Rødland EA, Staerfeldt HH, Rognes T, Ussery DW. RNAmmer: consistent and rapid annotation of ribosomal RNA genes. Nucleic Acids Res. 2007;35:3100–8.

10. Edgar RC. MUSCLE: multiple sequence alignment with high accuracy and high throughput. Nucleic Acids Res. 2004;32:1792–7.

11. Kearse M, Moir R, Wilson A, Stones-Havas S, Cheung M, Sturrock S, et al. Geneious Basic: an integrated and extendable desktop software platform for the organization and analysis of sequence data. Bioinformatics. 2012;28:1647–9.

12. Suyama M, Torrents D, Bork P, Delbru M. PAL2NAL: robust conversion of protein sequence alignments into the corresponding codon alignments. Nucleic Acids Res. 2006;34:609–12.

13. Nylander JA. Nylander/catfasta2phyml. https://github.com/nylander/catfasta2phyml

14. Huson DH, Bryant D. Application of phylogenetic networks in evolutionary studies. Mol Biol Evol. 2006;23:254–67.

15. Stamatakis A. RAxML-VI-HPC: maximum likelihood-based phylogenetic analyses with thousands of taxa and mixed models. Bioinformatics. 2006;22:2688–90.

16. Bruen TC, Philippe H, Bryant D. A simple and robust statistical test for detecting the presence of recombination. Genetics. 2006;172:2665–81.

17. Marttinen P, Hanage WP, Croucher NJ, Connor TR, Harris SR, Bentley SD, et al. Detection of recombination events in bacterial genomes from large population samples. Nucleic Acids Res. 2012;40:e6.

18. Didelot X, Wilson DJ. ClonalFrameML: efficient inference of recombination in whole bacterial genomes. PLOS Comput Biol. 2015;11:e1004041.

19. Lanza VF, de Toro M, Garcillan-Barcia MP, Mora A, Blanco J, De Cruz F, et al. Plasmid flux in *Escherichia coli* ST131 sublineages, analyzed by Plasmid Constellation Network (PLACNET), a new method for plasmid reconstruction from whole genome sequences. PLoS Genet. 2014;10:e1004766.

20. Langmead B, Salzberg SL. Fast gapped-read alignment with Bowtie 2. Nat Methods. 2012;9:357–9.

21. Picard-tools 1.9. https://sourceforge.net/projects/picard/files/picard-tools/

22. NCBI ftp site. ftp://ftp.ncbi.nlm.nih.gov/

23. Smoot ME, Ono K, Ruscheinski J, Wang PL, Ideker T. Cytoscape 2.8: new features for data integration and network visualization. Bioinformatics. 2011;27:431–2.

Technical Appendix Table. Isolates examined in the current study

Identifier	Species/serogroup	Date*	Source	Country	Linked to†
02.4755	*L. anisa*	Sep 24, 2002	Hot water supply	Scotland	-
03.5252	*L. anisa*	Nov 12, 2003	Patient	Scotland	-
04.2845	*L. longbeachae* Sg1	Jun 7, 2004	Patient	Scotland	-
06.3325	*L. anisa*	Jul 18, 2006	Patient	Scotland	-
08.1921	*L. longbeachae* Sg1	Apr 1, 2008	Patient	Scotland	08.2077,08.2078
08.2077	*L. longbeachae* Sg1	Apr 17, 2008	Compost-IMS	Scotland	08.1921
08.2078	*L. longbeachae* Sg1	Jul 31, 2008	Compost Direct	Scotland	08.1921
09.5279	*L. longbeachae* Sg1	May 20, 2009	Patient	Scotland	09.5470–4
09.5470–4	*L. longbeachae* Sg2	Jun 2, 2009	Compost-Direct	Scotland	09.5279
09.6863	*L. longbeachae* Sg1	Nov 19, 2009	Compost	Scotland	Patient negative
10.4571	*L. longbeachae* Sg1	Mar 19, 2010	Patient	Scotland	Compost negative
11.3483(3)	*L. longbeachae* Sg1	May 13, 2011	Compost	Scotland	11.3484(1)
11.3484(1)	*L. longbeachae* Sg1	May 13, 2011	Compost	Scotland	11.3483(3)
12.4709	*L. longbeachae* Sg1	Jun 18, 2012	Patient	Scotland	No compost
13.8641	*L. longbeachae* Sg1	Jun 18, 2012	Compost	Scotland	13.8644/45
13.8642	New species	Jun 18, 2012	Compost	Scotland	13.8643
13.8643	*L. longbeachae* Sg1	Jun 18, 2012	Compost	Scotland	13.8642
13.8644	*L. longbeachae* Sg1	Jun 18, 2012	Compost	Scotland	13.8641/45
13.8645	*L. longbeachae* Sg1	Jun 18, 2012	Compost	Scotland	13.8641/44
13.8646	*L. longbeachae* Sg1	Jun 26, 2013	Compost	Scotland	-
13.4628	*L. longbeachae* Sg1	Jun 26, 2013	Compost	Scotland	-
13.4630	*L. longbeachae* Sg1	Aug 23, 2013	Compost	Scotland	-
13.5970‡	*L. longbeachae* Sg1	Aug 23, 2013	Patient	Scotland	13.6038
13.59701‡	*L. longbeachae* Sg1	Aug 23, 2013	Patient	Scotland	13.6038
13.59702‡	*L. longbeachae* Sg1	Aug 23, 2013	Patient	Scotland	13.6038
13.59703‡	*L. longbeachae* Sg1	Aug 23, 2013	Patient	Scotland	13.6038
13.59704‡	*L. longbeachae* Sg1	Aug 27, 2013	Patient	Scotland	13.6038
13.6038‡	*L. longbeachae* Sg1	Aug 30, 2013	Top soil	Scotland	13.5970
13.6121‡	*L. longbeachae* Sg1	Aug 30, 2013	Patient	Scotland	13.6619/27
13.61211‡	*L. longbeachae* Sg1	Aug 30, 2013	Patient	Scotland	13.6619/27
13.61212‡	*L. longbeachae* Sg1	Aug 30, 2013	Patient	Scotland	13.6619/27
13.61214‡	*L. longbeachae* Sg1	Sep 6, 2013	Patient	Scotland	13.6619/27
13.6310‡	*L. longbeachae* Sg1	Sep 6, 2013	Patient	Scotland	13.6762
13.6314‡	*L. longbeachae* Sg1	Sep 13, 2013	Patient	Scotland	13.6619
13.6472‡	*L. longbeachae* Sg1	Sep 13, 2013	Patient	Scotland	No compost
13.64721‡	*L. longbeachae* Sg1	Sep 13, 2013	Patient	Scotland	No compost
13.64722‡	*L. longbeachae* Sg1	Sep 13, 2013	Patient	Scotland	No compost
13.64723‡	*L. longbeachae* Sg1	Sep 17, 2013	Patient	Scotland	No compost
13.64724‡	*L. longbeachae* Sg1	Sep 19, 2013	Patient	Scotland	No compost
13.6557‡	*L. longbeachae* Sg1	Sep 19, 2013	Patient	Scotland	No compost
13.6614‡	*L. longbeachae* Sg1	Sep 19, 2013	Compost	Scotland	-
13.6619‡	*L. longbeachae* Sg1	Sep 20, 2013	Compost	Scotland	13.6121
13.6627‡	*L. longbeachae* Sg1	Sep 25, 2013	Compost	Scotland	13.6121
13.6634‡	*L. longbeachae* Sg1	Sep 25, 2013	Patient	Scotland	No compost
13.6762‡	*L. longbeachae* Sg1	Sep 25, 2013	Compost	Scotland	13.6310
13.6763‡	*L. longbeachae* Sg1	Oct 1, 2013	Compost	Scotland	-
13.6764‡	*L. longbeachae* Sg1	Oct 1, 2013	Compost	Scotland	-
13.6912	*L. longbeachae* Sg1	May 29, 2014	Compost	Scotland	-
13.6914	*L. longbeachae* Sg1	May 7, 2014	Compost	Scotland	-
8702918	*L. longbeachae* Sg1	May 8, 2014	Patient	Scotland	870286x
8702860	*L. longbeachae* Sg1	May 9, 2014	Soil	Scotland	8702918
8702861	*L. longbeachae* Sg1	May 10, 2014	Soil	Scotland	8702918
8702862	*L. longbeachae* Sg1	Apr 1, 2008	Soil	Scotland	8702918
8702863	*L. longbeachae* Sg1	Apr 17, 2008	Soil	Scotland	8702918
13.8292	*L. anisa*	2010	Compost	New Zealand	13.8293

Identifier	Species/serogroup	Date*	Source	Country	Linked to†
13.8293	*L. longbeachae* Sg1	2010	Patient	New Zealand	13.8292
13.8294	*L. longbeachae* Sg1	2004	Patient	New Zealand	-
13.8295	New species	2013	Patient	New Zealand	-
13.8296	*L. longbeachae* Sg2	2012	Sump drain	New Zealand	-
13.8297	*L. longbeachae* Sg2	2007	Compost	New Zealand	13.8301
13.8298	*L. longbeachae* Sg1	1996	Compost	New Zealand	-
13.8299	*L. longbeachae* Sg1	2003	Compost	New Zealand	-
13.8300	*L. longbeachae* Sg2	2011	Compost	New Zealand	-
13.8301	*L. longbeachae* Sg2	2007	Patient	New Zealand	13.8297
Sg2	*L. longbeachae* Sg2	-	-	-	-
NSW 150	*L. longbeachae* Sg1	-	Patient	Australia	-
C-4E7	*L. longbeachae* Sg2	-	Patient	Australia	-
D-4968	*L. longbeachae* Sg1	-	Patient	USA	-
ATCC39642	*L. longbeachae* Sg1	-	Patient	USA	-
98072	*L. longbeachae* Sg2	-	Patient	USA	-

*Date received in the reference laboratory.

†Isolates that are linked to each other, as patient and cognate compost samples. Multiple isolates from each of the three patients or environmental samples share common identifiers (13.5970, 13.6121, 13.6472, 870286).

‡Isolates from the 2013 summer cluster of diseases in Scotland.

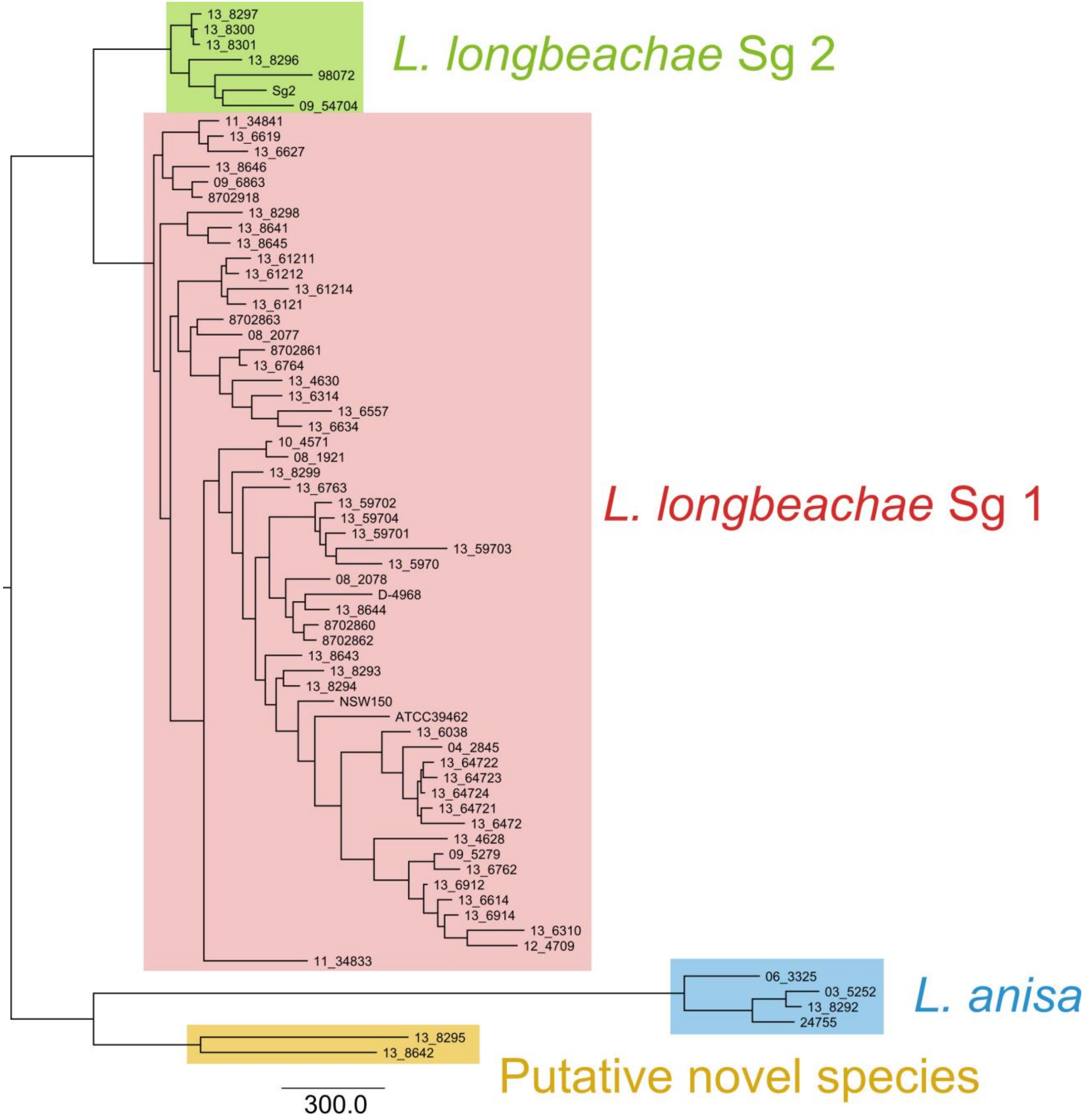

Technical Appendix Figure 1. Parsimony based tree of the OMCL pangenomic matrix obtained for all the sequenced genomes. Scale bar indicates the gene content differences.

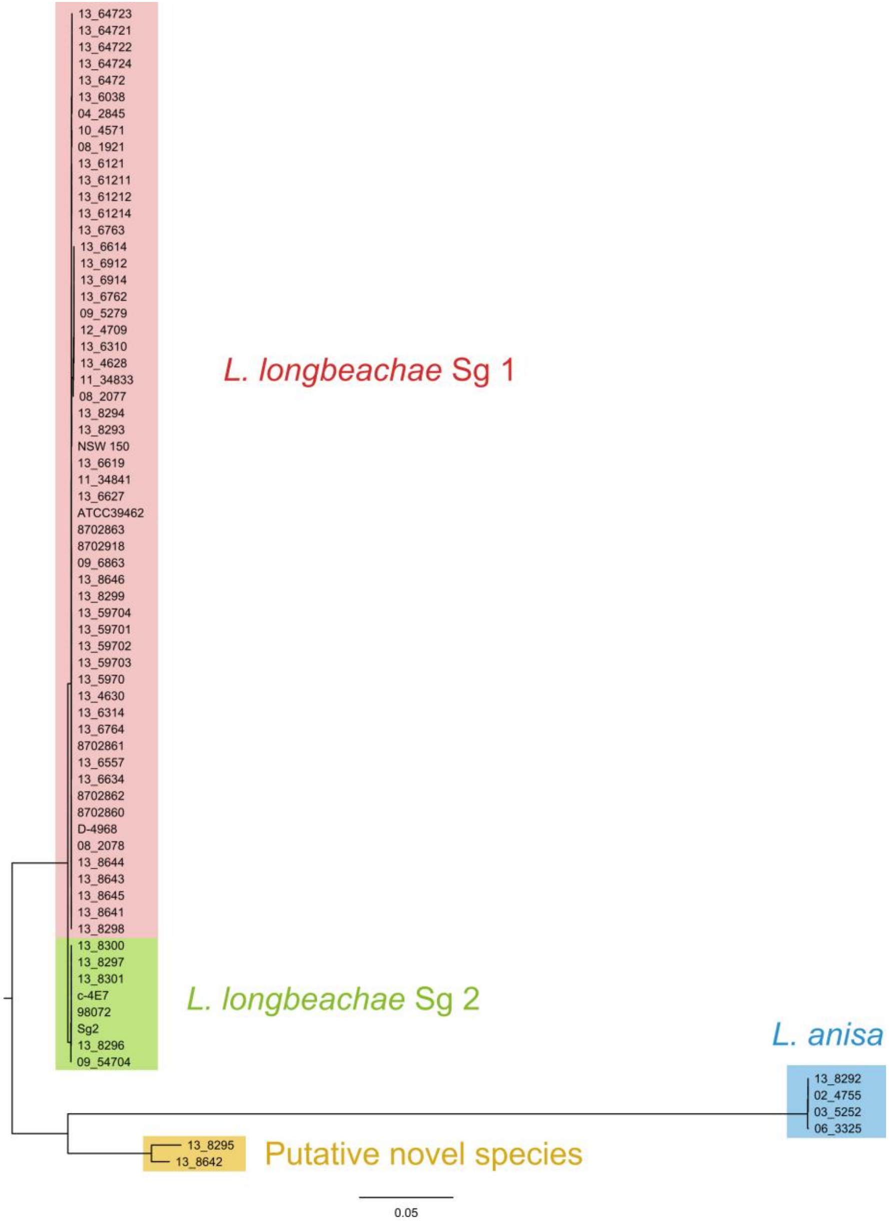

Technical Appendix Figure 2. Maximum likelihood tree of a core gene alignment of all the isolates included in the study. The tree shows the same clusters as the 16S rRNA gene based tree and the parsimony pangenome tree. Scale bar indicates the mean number of nucleotide substitutions per site.

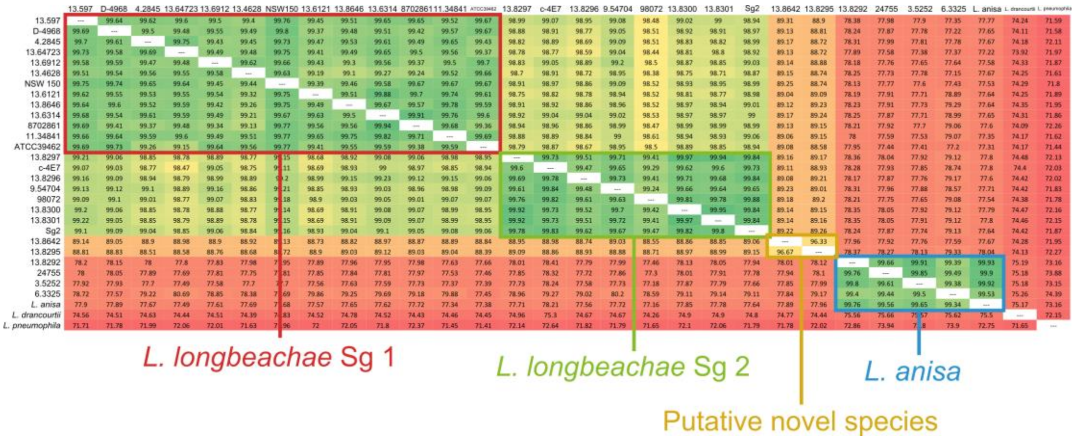

Technical Appendix Figure 3. Average Nucleotide Identity (ANI) comparison matrix of several genomes of the study sorted by similarity. Green, high ANI values; Red, low ANI values. The genomes of *Legionella drancourtii* and *Legionella pneumophila* were included in the matrix for comparative purposes, but none of the isolates showed an ANI higher than 76% with these two species.

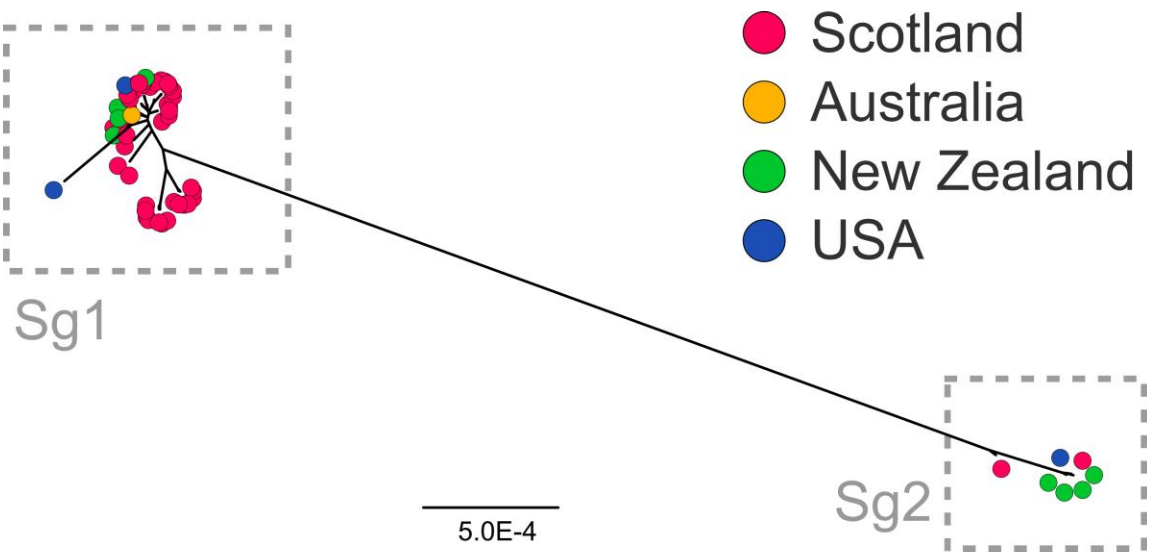

Technical Appendix Figure 4. Neighbor-Joining phylogeny based on the core genome of *Legionella longbeachae* isolates. Isolates are colored by geographic source, and dashed boxes indicate the defined or predicted serogroups to which the isolates belong.

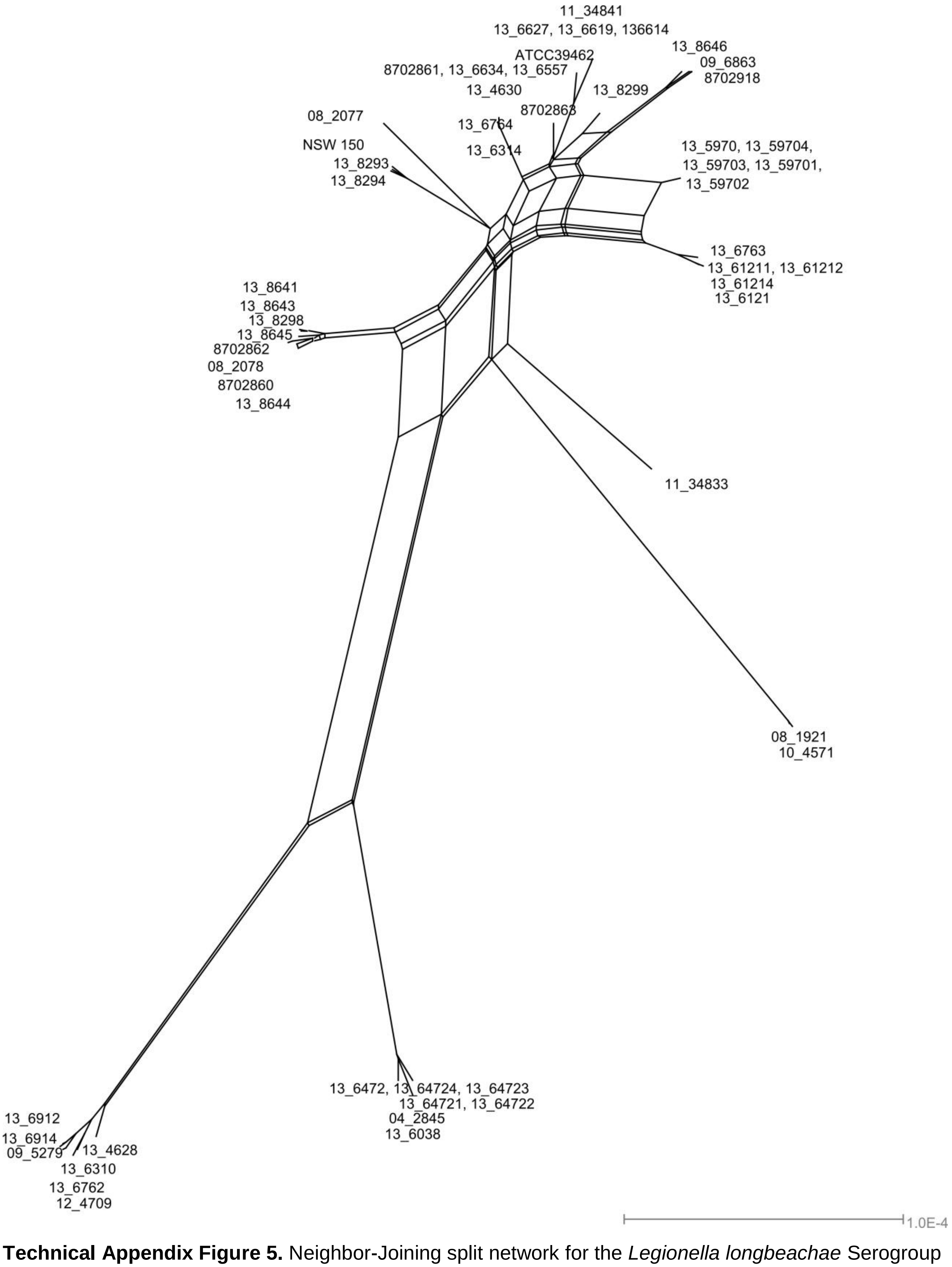

Technical Appendix Figure 5. Neighbor-Joining split network for the *Legionella longbeachae* Serogroup 1 isolates based on the consensus alignment obtained from mapping every isolate to the reference chromosome NSW 150. Scale bar indicates the mean number of nucleotide substitutions per site.

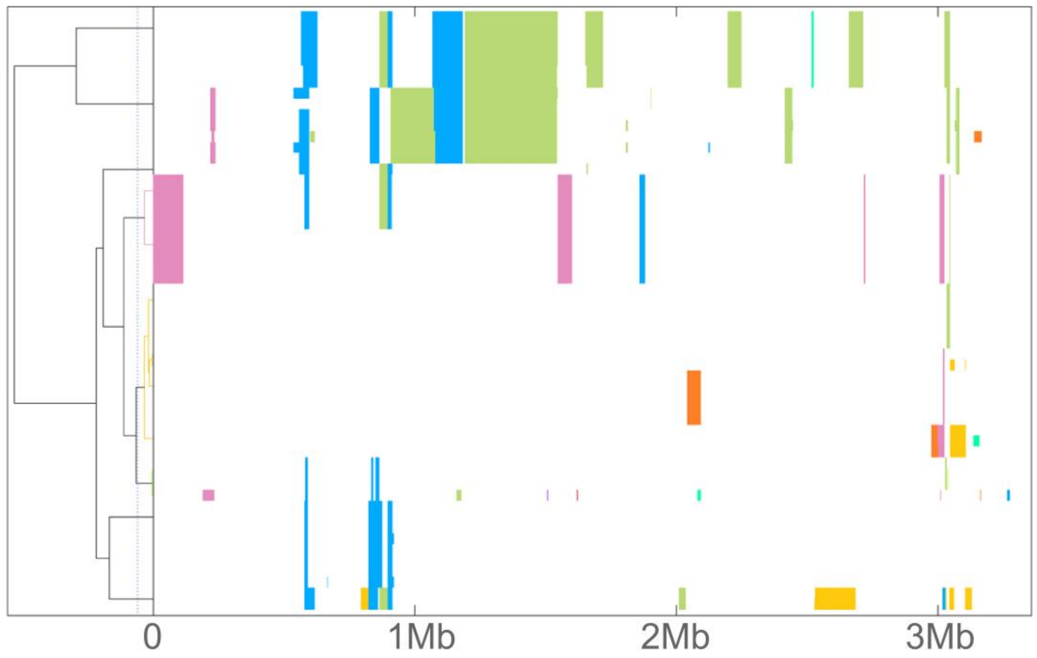

Technical Appendix Figure 6. Recombinant regions of the core genome alignment of 55 *L. longbeachae* Sg1 isolates as identified using BratNextGen. On the left, a clustering tree of the isolates with colored branches indicating cluster relationships. On the right, significant recombinant segments predicted, with similar color in a column representing recombinant regions for those isolates have the same origin.
